# A case of transplantation-associated thrombotic microangiopathy with autopsy-proven fatal myocardial ischemia after allogeneic hematopoietic stem cell transplantation

**DOI:** 10.1007/s00277-020-04027-0

**Published:** 2020-05-06

**Authors:** Ken Sasaki, Akira Honda, Aya Shinozaki-Ushiku, Yosei Fujioka, Hiroaki Maki, Kazuhiro Toyama, Tetsuo Ushiku, Mineo Kurokawa

**Affiliations:** 1 Department of Hematology and Oncology, Graduate School of Medicine, The University of Tokyo, 7-3-1 Hongo, Bunkyo-ku, Tokyo, 113-8655 Japan; 2 Department of Pathology, Graduate School of Medicine, The University of Tokyo, 7-3-1 Hongo, Bunkyo-ku, Tokyo, 113-8655 Japan; 3 Department of Cell Therapy and Transplantation Medicine, The University of Tokyo Hospital, 7-3-1 Hongo, Bunkyo-ku, Tokyo, 113-8655 Japan

Dear Editor,

Hematopoietic stem cell transplantation-associated thrombotic microangiopathy (TA-TMA) is one of the most severe complications after allogeneic hematopoietic stem cell transplantation (HSCT) [1, 2]. Although TA-TMA commonly affects kidneys, lungs, and the gastrointestinal tract, only one case of TA-TMA with cardiac involvement has been reported. In this report, limited cardiac involvement was proven by autopsy, but it was not the main cause of death [3]. Herein, we report a first case of fatal myocardial ischemia caused by TA-TMA proven by autopsy.

A 59-year-old female underwent allogeneic HSCT from an HLA 8/8 matched unrelated donor with non-myeloablative conditioning for AML. She developed chronic graft-versus-host disease 9 months after transplant. During the treatment with prednisolone and cyclosporin (CyA), she was diagnosed with TA-TMA based on the findings of thrombocytopenia (7.4 × 10^4^ /μL), anemia (10.3 g/dL), schistocytes (20–25/HPF), elevated lactate dehydrogenase (LDH) (874 IU/L), and decreased haptoglobin (3 mg/dL). Despite the cessation of CyA, she noticed the exacerbation of the shortness of breath and the edema of both legs. Echocardiography showed reduced ejection fraction (EF 23%) and global hypokinesis. Right ventricular systolic pressure (RVSP) was not elevated (23 mmHg). The blood tests showed the elevated troponin T (0.227 ng/mL) and the elevated BNP (1770.6 pg/mL), but did not show the elevation of creatine kinase (CK 108 U/L) or CK-MB (12 U/L). Electrocardiogram showed inverted T waves in leads I, II, and V3–6. Coronary angiography and biopsy could not be done because of severe thrombocytopenia. Then, her cardiac dysfunction was exacerbated, and she died after sudden cardiac arrest despite the treatment.

**Fig. 1:**
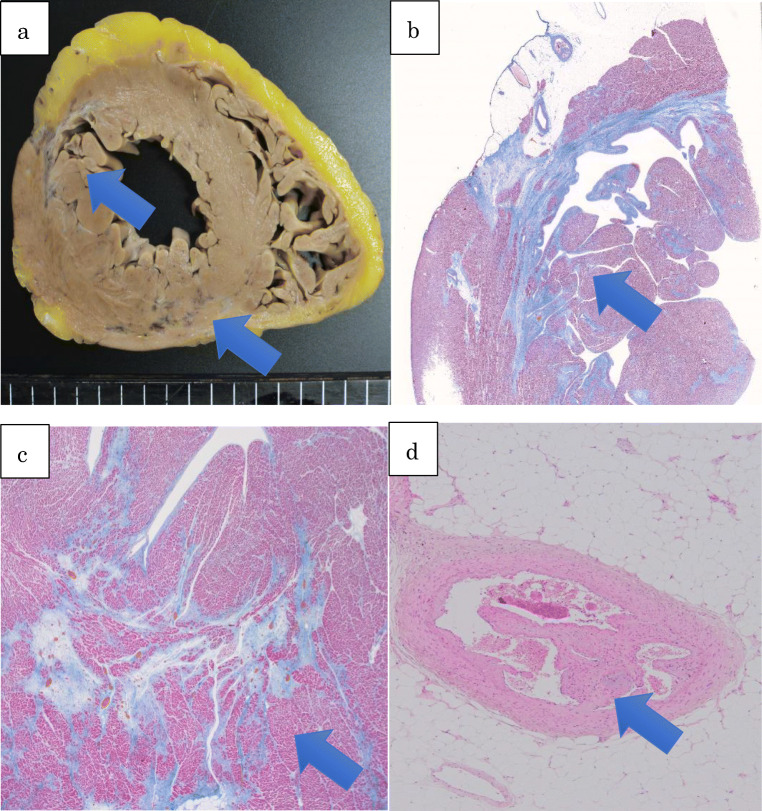
**a** Macroscopic examination reveals fibrosis on the anterior and posterior wall of the left ventricle. **b**, **c** Microscopic examination reveals fibrosis on the anterior and posterior wall of the left ventricle. **d** Microscopic examination reveals the organized thrombus in the distal part of the left anterior descending artery

An autopsy revealed the dilation of the left ventricle and fibrosis on the anterior and posterior wall of the left ventricle of the heart (Fig. 1a). Microscopic examination revealed fibrosis with edema and dilated capillaries on the anterior, posterior, and lateral walls. It also showed the intimal thickening and the narrowing of the lumen of the arterioles and the organized thrombus in the distal part of the left anterior descending artery, which suggest the involvement of TA-TMA (Fig. 1b–d). Based on these findings, we concluded that the cause of death was myocardial ischemia due to TA-TMA. There were no signs of relapse of AML.

Cardiac complications after HSCT have been reported previously, and they are most likely to be caused by the conditioning regimens [4, 5]. Only one case of cardiac involvement of TA-TMA has been reported, in which TA-TMA was not the main cause of death [3]. In the present case, we experienced a first case of fatal myocardial ischemia caused by TA-TMA. As the present case suggests, myocardial ischemia caused by TA-TMA may not be reversible by the withdrawal of calcineurin inhibitors even when other findings including renal failure and hemolysis improve. In conclusion, regular cardiac evaluation including echocardiography, ECG, and cardiac enzymes are recommended after HSCT, and TA-TMA should be considered in patients with reduced EF after HSCT. Further cases are warranted to find the best management of myocardial ischemia caused by TA-TMA.
